# A Fresh Breath of Oxygen: Red Blood Cell Exchange Transfusion in Sickle Cell and COVID‐19

**DOI:** 10.1002/ccr3.4655

**Published:** 2021-08-24

**Authors:** Viva Nguyen, Paul Alcius, Shachar Peles, Katherine Hodgin

**Affiliations:** ^1^ University of Miami JFK GME Atlantis FL USA; ^2^ Florida Cancer Specialists & Research Institute Lake Worth FL USA; ^3^ JFK Medical Center Atlantis FL USA

**Keywords:** acute chest syndrome, COVID‐19, hereditary persistence of fetal hemoglobin, red blood cell exchange transfusion, sickle cell disease

## Abstract

Red blood cell exchange transfusion may be beneficial and should be considered in the early management of patients with sickle cell disease and COVID‐19 to prevent the need for intubation and intensive care unit admission due to respiratory distress.

## Graphical abstract

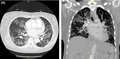

## 1 INTRODUCTION

We present the first documented case of a 69‐year‐old female with a history of sickle cell disease with hereditary persistence of fetal hemoglobin that presented in sickle cell crisis and COVID‐19 infection. The red blood cell exchange transfusion may play a role in preventing intubations and longer hospital courses.

Sickle cell disease (SCD) is one of the most common hemoglobinopathies in the world, with important complications such as vaso‐occlusive crisis (VOC) and acute chest syndrome (ACS). With the worldwide pandemic of COVID‐19, the overlap between clinical presentations of life‐threatening pneumonia versus ACS has blurred the lines for treatment. Early treatment with red blood cell exchange transfusion in patients with SCD, as well as others hemoglobinopathies, may be a key factor in improving outcomes, including the prevention of respiratory failure requiring mechanical ventilation, in those with COVID‐19 infection.

## 2 CASE REPORT

**FIGURE 1:**
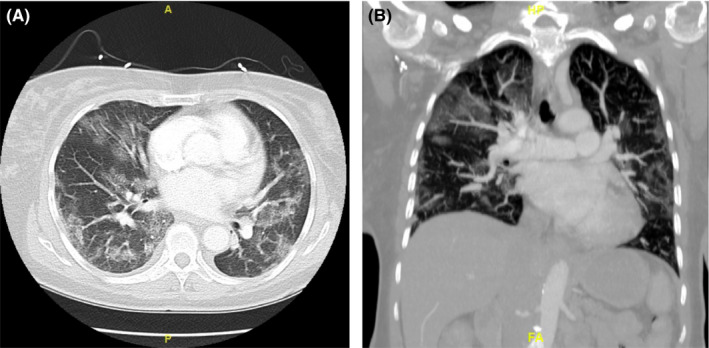
Computed tomography angiogram of the chest: diffuse bilateral subpleural ground‐glass opacities are seen consistent with the reported diagnosis of COVID pneumonia

A 69‐year‐old African American female with a past medical history of sickle cell disease with mild phenotype from hereditary persistence of fetal hemoglobin (HPFH) with baseline hemoglobin S fraction of 75% on folic acid presented to the emergency department (ED) due to joint pain and fevers of 2‐day duration. On admission, she denied any chest pain, shortness of breath, cough, nausea, vomiting, and diarrhea. The patient reported that she tested positive for COVID‐19 at an outpatient facility a day prior to admission with no known prior exposure. She was hemodynamically stable, afebrile with oxygen saturation of 95% on room air. Physical examination was notable for bilateral crackles. Laboratory studies demonstrated a positive COVID‐19 infection via a rapid polymerase chain reaction with elevated inflammatory markers of D‐dimer >5250 ng/mlDDU (normal 0–316 ng/mlDDU), lactate dehydrogenase 324 units/L (normal 84–246 units/L), C‐reactive protein 1.3 mg/dl (normal 0–1.0 mg/dl), and a hemoglobin of 6.4 g/dl (normal 11.2–15.7 g/dl). Chest X‐ray (CXR) upon admission demonstrated mild perihilar interstitial infiltrates with mild peripheral right ground‐glass infiltrates. Computed tomography (CT) angiogram of the chest was performed revealing extensive bilateral ground‐glass subpleural opacity with no evidence of pulmonary emboli (Figure 1). A unit of packed red blood cells (pRBCS) was ordered and transfused in the ED. The patient was admitted for sickle cell crisis, symptomatic anemia, and COVID‐19 pneumonia. She was immediately started on ceftriaxone, azithromycin, convalescent plasma, remdesivir, dexamethasone, zinc, vitamin C, and vitamin D with infectious disease consulted.

Two days after admission, the rapid response team was called due to acute shortness of breath. Upon initial evaluation, the patient was in respiratory distress with use of accessory muscles. Pulse oximeter revealed an oxygen saturation of 87% on nonrebreather (NRB). At that time, the decision was made to transfer the patient to the intensive care unit (ICU) for planned red blood cell exchange transfusion (RBCX) to prevent further respiratory failure. Upon arrival to the ICU, the patient's respiratory status continued to decline. Heart rate was 160–170 beats per minute and oxygen saturation was 65%–70% on NRB; the patient was tachypneic and severely agitated. As a result, the patient was sedated, intubated, and placed in prone position for 16 h. The patient's arterial blood gas (ABG) following intubation showed pH 7.287, PCO_2_ 30.0 mmHg, PO_2_ 318.4 mmHg, and HCO_3_ 14.2 mM/L with P/F ratio of 318.40 on FiO_2_ of 100%. Ten units of hemoglobin S negative, leukocyte reduced RBCs were ordered and transfused throughout the night. Hemoglobin electrophoresis following the RBCX demonstrated: hemoglobin A 92.1%, hemoglobin A2 2.5%, hemoglobin C 0.0%, hemoglobin F 0.0%, and hemoglobin S 5.4%. As a result of the RBC transfusion exchange, another unit of convalescent plasma was transfused the following day. The ABG on the following day revealed pH 7.487, PCO_2_ 28.3 mmHg, PO_2_ 144.8 mmHg, and HCO_3_ 21.0 mM/L with P/F ratio of 289.60 on FiO2 of 50%. Ultimately, the patient was extubated 2 days after intubation and transferred out of the ICU the following day after extubation.

## 3 DISCUSSION

The patients with SCD often present to the hospital because of acute pain or symptomatic anemia. Hemoglobin is normally soluble in erythrocytes and does not polymerize; hemoglobin S (HbS), resulting from a point mutation in the beta globulin gene, becomes poorly soluble when deoxygenated.^1^ This pathological polymerization of HbS, which forms the classic crescent or sickle shape of erythrocytes, is one of the main causes of VOC.^1^ The degree of polymerization is a determinant of the severity of SCD, which is affected by other hemoglobin mutations co‐occurring with HbS and concentration of fetal hemoglobin.

An important factor in the clinical manifestation and prognosis of our 69‐year‐old patient is the presence of hereditary persistence of fetal hemoglobin (HPFH). Uniquely, our patient was homozygous for HPFH, which likely contributed to the longevity of her lifespan. The patients with SCD are immunocompromised, thus avoiding COVID‐19 is crucial.

Like any other respiratory viruses, COVID‐19 may cause pneumonia, leading to potential ACS or respiratory failure in those with SCD.^2^ Because symptoms overlap between COVID‐19 pneumonia and ACS, any potential deterioration in respiratory status needs prompt intervention. There have been cases reported of COVID‐19 causing ACS.^3^ It is hypothesized that a possible mechanism of severe acute respiratory syndrome coronavirus 2 (SARS‐CoV‐2) is that the virus affects the normal, functioning structure of hemoglobin, causing the hypoxemia.^4^ Thus, by directly addressing the abnormal hemoglobin with RBCX, we may prevent rapid declines in oxygenation.

In our patient's situation, her respiratory status deteriorated rapidly to a point where intubation was deemed necessary due to refractory hypoxemia and to allow her body to rest while receiving the RBCX. Studies have suggested that exchange transfusion is superior to simple blood transfusion in respiratory failure and multi‐organ failure.^5^ The goal of an exchange transfusion is to significantly lower HbS levels to 30 percent or less and correct any potential anemia, which was demonstrated by the patient's hemoglobin electrophoresis following the exchange. By lowering the HbS level, in particular, those with acute organ deterioration, we limit the likelihood of sickling, as well as its complications.

For our patient, she got intubated and then received the RBCX. The patient recovered rather quickly after intubation, just requiring 48 h on the mechanical ventilator, which is highly uncommon for those with COVID‐19 pneumonia and its complications. The RBCX improved her oxygenation status and ultimately led to a rapid recovery. In other case reports, there have been suggestions of conducting RBCX in patients with SCD and COVID‐19 after admission and before rapid respiratory deterioration to prevent intubation and ICU admission.^4^, ^6^, ^7^, ^8^ Our patient had to be intubated prior to undergoing a RBCX. Perhaps if we were able offer the patient RBCX prior to her acute onset of respiratory distress, we could have prevented the mechanical ventilation, ICU admission, and the extra dose of convalescent plasma, which are certainly costly in the hospital setting.

Limitations for our proposed prophylactic RBCX prior to respiratory deterioration include limited reported cases, in particular, those patients with SCD and COVID‐19. Likewise, blood bank shortages may prevent the possibility of RBCX at a given facility. The transfer of potential patients to facilities that could conduct an RBCX could be timing consuming and costly as well.

## 4 CONCLUSION

This case report demonstrates that a well‐known procedure of RBCX should be considered in the early management of patients with SCD and COVID‐19 to prevent the need for mechanical ventilation and ICU admission due to respiratory distress. RBCX appears to be beneficial not only to the patient's overall health but also to the healthcare system by reducing cost as well as limiting the use of sparse resources, such as ICU beds and staffing, during a worldwide pandemic. Further studies will need to be conducted to validate benefits of early RBCX in SCD and COVID‐19 patients.

## CONFLICTS OF INTEREST

The authors declare that they have no conflicts of interest.

## AUTHOR CONTRIBUTIONS

VN analyzed and interpreted the patient data regarding the patient's hospital course and wrote the majority of the foundation of the case report. PA reanalyzed the data and made major edits to the initial draft. SP gave input in regard to the hematological process. KH gave input in regard to the acute pulmonary and intensive care course. All authors read and approved the final manuscript.

## CONSENT

Written informed consent for patient information and images was provided by the patient.

## Data Availability

The data that support the findings of this study are available from the corresponding author upon reasonable request.
